# Prognostic Value of Antiarrhythmic Drug Suppression of Electrical Storm Prior to Ventricular Tachycardia Ablation

**DOI:** 10.1111/jce.70133

**Published:** 2025-10-15

**Authors:** Rafik Issa, Osama El‐Sayed, Mohammed Al‐Sadawi, Muazzum Shah, Kelly Arps, Amrish Deshmukh, Michael Ghannam, Rakesh Latchamsetty, Krit Jongnarangsin, Thomas Crawford, Aman Chugh, Hakan Oral, Fred Morady, Frank Bogun, Jackson J. Liang

**Affiliations:** ^1^ Department of Internal Medicine University of Michigan Ann Arbor Michigan USA; ^2^ Department of Cardiovascular Medicine University of Michigan Ann Arbor Michigan USA

**Keywords:** ablation, antiarrhythmic drugs, electrical storm, ventricular tachycardia

## Abstract

**Introduction:**

Early ablation after electrical storm (ES) has been associated with improved ventricular tachycardia (VT)‐free survival. Antiarrhythmic drugs (AAD) can acutely temper ES in some patients allowing for delayed elective ablation, but they may be ineffective in other patients, who may require urgent ablation. The prognostic impact of acute AAD efficacy for ES patients undergoing ablation is unclear and may help to inform timing of VT ablation. The purpose of our study is to compare the outcomes of patients with ES undergoing VT ablation based on acute AAD efficacy.

**Methods:**

This retrospective study of patients with ES who underwent VT ablation at our institution between November 2018 and September 2023 compared those who underwent urgent ablation due to inefficacy of AAD (Urgent) versus those in whom AAD controlled ES acutely and ablation could be performed in an elective manner (Elective). The timing of ablation was based on provider discretion and ability to control ES on AAD. Long term survival, VT‐free survival, ES‐free survival and repeat ablation‐free survival were compared through Kaplan Meir analysis and the log‐rank test. Individual predictors of survival and VT‐free survival were identified through Cox proportional hazards model with univariate and multivariate regression analysis.

**Results:**

One hundred and twenty patients were included (*n* = 68 urgent, median 7 days postepisode of ES vs. *n* = 52 elective, median 86 days post ES). Major complications were significantly higher in the urgent group (17.7% vs. 3.9%, *p* = 0.023). The Elective group had significantly improved long‐term survival at time of follow up (*χ* ^2^ = 7.4, *p* = 0.0065). There were no significant differences in VT‐free, ES‐free survival or repeat ablation‐free survival. Cox multivariate regression indicated significantly increased mortality in the Urgent group (*p* = 0.039), but no difference in VT recurrence (*p* = 0.88). An increased number of inducible VT foci during ablation was significantly associated with increased mortality. Use of B‐blockers was associated with decreased rates of VT recurrence.

**Conclusion:**

Patients with ES who were able to be electrically stabilized with AAD and returned for an elective ablation had improved survival compared to those who required urgent ablation, although there were no differences in VT and ES recurrence rates. Stabilization with AAD before VT ablation is a positive prognostic factor for survival in ES.

## Graphical abstract

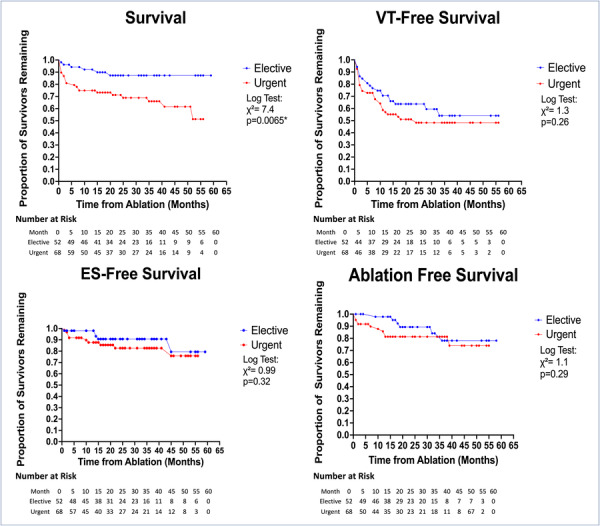

## 1 Introduction

Electrical storm (ES) refers to a syndrome of recurrent episodes of sustained ventricular arrhythmia (VA), defined as three or more episodes of sustained VA separated by at least 5 min in a 24‐h period [1]. ES is associated with increased mortality and HF hospitalizations; as low as 2 episodes of VA in 3 months is associated with a significantly worse prognosis [2, 3, 4]. The VA is most often monomorphic ventricular tachycardia (VT) but also encompasses polymorphic VT and ventricular fibrillation (VF) [5, 6].

The management of ES is multifaceted and can be divided into acute and subacute or chronic phases [7, 8]. Within the acute phase, there is an established role for antiarrhythmic drugs (AAD) including amiodarone, lidocaine, sotalol and procainamide in terminating the ES event [9, 10, 11, 12, 13, 14, 15]. Following the immediate stabilization stage, ES management centers around preventing subsequent VA recurrences or reducing the VA burden. In this domain, AAD are frequently used for long‐term suppression of VA. Sympathetic blockade in various forms, including deep sedation, stellate ganglion blocks, thoracic epidurals and surgical sympathectomy, has been employed to suppress VA [16, 17, 18, 19, 20, 21, 22]. Catheter ablation is an increasingly utilized therapy for the definitive management of VA and ES in both ischemic and nonischemic cardiomyopathy (NICM) patients [23, 24, 25, 26, 27, 28, 29, 30]. There are no randomized controlled trials comparing early vs delayed ablation at time of ES. However, there is observational evidence that early referral for catheter ablation in VT is associated with improved VT‐free survival and procedural success [31, 32, 33]. A recent study by Huang et al. focused on ES specifically and concluded that early catheter ablation compared to initial medical therapy improves VT‐free survival [34]. However, in some situations, AADs may be attempted first to suppress ES and allow for later elective procedure. Such circumstances include when patients are felt to be at high risk for ablation due to current conditions at time of ES, when additional preprocedural imaging such as cardiac computed tomography angiography (CTA) or cardiac magnetic resonance imaging (MRI) would ideally be performed for planning purposes, or if there is lack of laboratory space or qualified VT ablationist availability to perform urgent ablation. The prognostic impact of acute AAD efficacy for ES patients undergoing ablation is unclear and may help to inform timing of VT ablation. For that reason, this study compares outcomes of patients with ES undergoing VT ablation based on acute AAD efficacy.

## 2 Methods

### 2.1 Design

This retrospective study of patients with ES who underwent VT ablation compares those who required urgent ablation due to inefficacy of AAD versus those in whom AAD controlled ES and ablation could be performed in an elective manner. A patient was considered stabilized if an oral regimen of AAD was effectively able to suppress recurrent VTs to facilitate hospital discharge. Patients who were stabilized were counted as “Elective,” whereas those who were unable to be stabilized were classified as “Urgent.” Timing of ablation was based on provider discretion and ability to control ES on AADs. Patients who were stabilized with AAD and were deemed stable for hospital discharge from a VA standpoint but remained admitted for other non‐VA reasons and ultimately underwent inpatient ablation were classified in the “Elective” group.

### 2.2 Data, Inclusion, and Exclusion Criteria

Our database consisted of all adult patients who underwent VT ablation at the University of Michigan between November 2018 and September 2023, amounting to 377 patients. Of those, we selected 140 patients who met the criteria for Electrical Storm, defined as three or more episodes of sustained VT separated by at least 5 min. We excluded 20 patients who did not have follow‐up after the procedure and patients whose ablation was performed > 12 months after their admission for ES. The final sample size included 120 patients with ES who underwent ablation within 1 year of their ES episode. Of note, we did not exclude patients with a prior episode of ES or a prior ablation. The study was approved by the Michigan Medicine Institutional Review Board.

### 2.3 Long Term Outcomes

The long‐term outcomes of interest included overall survival, VT‐free survival (where VT recurrence was defined as an episode of sustained VT or VF lasting > 30 s or resulting in hemodynamic instability), ES‐free survival, and repeat‐ablation free survival.

### 2.4 Immediate Procedural Outcomes

The immediate procedural outcomes of interest included recurrent VT in 24 h, recurrent VT in index hospitalization before discharge, death during index hospitalization before discharge, and procedural complications. Certain procedural complications were defined as major complications and analyzed accordingly. Major complications included cardiac arrest, death, cardiogenic shock, post‐procedural septic shock, postprocedural hypoxic respiratory failure, post‐procedural hemorrhagic shock, cardiac perforation, and complete AV block. Additionally, we compared acute procedural success rates between our two groups. Successful ablation was defined as termination of all inducible VTs, partial success was defined as termination of clinical VT but with some nonclinical VTs inducible at the end of the procedure, and unsuccessful ablation was defined as failure to terminate clinical VT.

### 2.5 Follow‐Up

Follow‐up was defined as time since the ablation procedure. Patients were generally monitored in a 3‐ and 12‐month post‐op visit with interrogations of their ICD in addition to home remote monitoring for device transmissions. For patients who followed outside of Michigan Medicine, review of clinical documentation was utilized.

### 2.6 Statistical Analysis

Statistical analysis was performed through the GraphPad Prism 10.4.1 software. Continuous data was represented by the mean and standard deviation for normal distributions, and with the median and interquartile range (IQR) for non‐normal distributions. Categorical variables were reported as percentages. For statistical comparison of baseline characteristics, procedural characteristics, and immediate post procedural outcomes, student's *T*‐Test was used for parametric datasets while Mann–Whitney *U* test was utilized for nonparametric data. Categorical variables were compared with Fischer's exact test. Survival, VT‐Free survival, ES‐Free survival and repeat ablation‐free survival were compared with the Kaplan Meier analysis and the log rank‐chi squared method. To determine the other predictors of survival and VT‐free survival, Cox proportional hazards model was employed for univariate and multivariate regression. Hazard ratios and two‐tailed *p* values are used to express the risk of mortality and VT recurrence. *p* < 0.05 was deemed significant.

### 2.7 Ablation Technique

All patients were sedated by the anesthesia team. Conscious sedation with monitored anesthesia care was prioritized in all patients. Sedation with benzodiazepine was generally avoided during the procedure. After vascular access was obtained, intravenous heparin was administered to maintain activated clotting time > 250 s. All patients underwent programmed ventricular stimulation with up to four extra stimuli performed from right ventricular sites before ablation to induce VT. Surface EKG was recorded in combination with bipolar intracardiac tracings and recorded on optical disk (Workmate Claris, Abbott Laboratories, Pleasanton, CA). A 3‐dimensional electroanatomic mapping system (CARTO, Biosense Webster, Diamond Bar, CA or Ensite, Abbott, Chicago, IL) was used in all patients. Mapping was performed either in point‐by‐point manner using an ablation catheter (ThermoCool ST, ThermoCool STSF, or QDOT, Biosense Webster, Diamond Bar, CA, or FlexAbility, TactiCath, TactiFlex, Abbott, Chicago, IL), or using a multielectrode mapping catheter (Optrell, Biosense Webster, Diamond Bar, CA, or Advisor HD Grid, Abbott, Chicago, IL). VT target sites were identified by either activation and entrainment mapping of VT if hemodynamically stable or with pace mapping and/or substrate mapping and targeted with ablation per operator's discretion. Post ablation programmed ventricular stimulation with up to four extra stimuli performed from right ventricular sites to assess for VT inducibility. The procedure was concluded once the patient was rendered non‐inducible or improved from baseline inducibility, per operator discretion. There were seven different attending ablation operators for this patient cohort.

## 3 Results

Of the 120 patients included in our study, 52 underwent elective ablation while 68 required urgent ablation. Baseline characteristics for both groups are presented in (Table 1). The median number of days of the ablation following ES in the elective group was 86 days, compared to 7 days in the urgent group. The baseline characteristics of our two groups were similar except for two variables. Mean BMI was slightly higher in the urgent group (31.4 vs. 29, *p* = 0.038). Additionally, six patients in the urgent group underwent a prior sympathectomy while none of the patients in the elective group did (*p *= 0.035). No patients in either group underwent stellate ganglion block or thoracic epidural placement. PAINESD scores before ablation were similar between groups. Seven patients (13.5%) in the elective group had a left ventricular assist device at baseline compared to five patients (7.4%) in the urgent group, (*p *= 0.36). While more patients in the elective group had a preoperative cardiac MRI and CT, the difference was not significant (*p *= 0.23 and *p* = 0.57 respectively). Additionally, there was no significant difference in the number of patients who were on two AAD before ablation (*p *= 0.55) (Supporting Information S1: Table 1) depicts the specific AAD in each group, with overall no significant differences in utilization of any specific AAD class. Furthermore, there were no differences in rates of holding AAD before ablation (Supporting Information S2: Table 2).

**Table 1:** Baseline characteristics.

Characteristic	Subcategory	Elective	Urgent	*p* value
Days from storm to ablation		86 (IQR 32–199)	7 (IQR 5–13)	< 0.001^a^
Age (years)		65 (IQR 53–72)	67 (IQR 59–72)	0.52
Gender				0.27
	Male	48 (92.3%)	57 (83.8%)	
	Female	4 (7.7%)	11 (16.2%)	
Body Mass Index		29 (s.d 5.9)	31.4 (s.d 6.1)	0.038^a^
Hypertension		42 (80.8%)	54 (79.4%)	> 0.99
Diabetes mellitus		18 (34.6%)	22 (32.4%)	0.85
Peripheral artery disease		2 (3.9%)	3 (4.4%)	0.99
Ischemic cardiomyopathy		32 (61.5%)	37 (54.4%)	0.46
Nonischemic cardiomyopathy		24 (46.1%)	33 (48.5%)	0.85
Chronic obstructive pulmonary disease		6 (11.5%)	10 (14.7%)	0.79
Chronic kidney disease, ≥ stage 3		12 (23.1%)	20 (29.4%)	0.53
Implantable cardiac defibrillator		52 (100%)	68 (100%)	0.99
Cardiac resynchronization therapy‐defibrillator		19 (36.5%)	33 (48.5%)	0.2
Atrial fibrillation		27 (51.9%)	43 (63.2%)	0.26
Left ventricular ejection fraction		31 (IQR 20‐49)	25 (IQR 20‐43)	0.096
Prior ventricular ablations		21 (40.4%)	28 (41.4%)	> 0.99
Prior sympathectomy		0 (0%)	6 (8.8%)	0.035^a^
Prior percutaneous coronary intervention		21 (40.4%)	32 (47.1%)	0.58
Prior coronary artery bypass graft		17 (32.7%)	15 (22.1%)	0.22
≥ Moderate aortic stenosis or regurgitation		3 (5.8%)	4 (5.9%)	0.99
≥ Moderate mital stenosis or regurgitation		4 (7.7%)	7 (10.3%)	0.76
Left ventricular assist device		7 (13.5%)	5 (7.4%)	0.36
History of prior arrest		7 (13.5%)	15 (22.1%)	0.24
On ≥ 2 antiarrhythmic drugs		34 (65.4%)	48 (71.6%)	0.55
On B‐blocker		46 (88.5%)	57 (83.8%)	0.59
Monomophic ventircular tachycardia present		48 (92.3%)	65 (95.6%)	0.47
Polymophic ventircular tachycardia present		6 (11.5%)	6 (8.8%)	0.76
Ventricular fibrillation present		3 (5.8%)	8 (11.8%)	0.35
PAINESD score		16.56 (s.d. 6.6)	17.28 (s.d. 6.6)	0.55
Cardiac MRI obtained pre‐op		39 (75%)	43 (63.2%)	0.23
Cardiac CT obtained pre‐op		34 (65.4%)	40 (58.8%)	0.57
Late gadolinium enhancement present on MRI		35 (89.7%)	35 (81.4%)	0.36
Wall thinning on CT		18 (52.9%)	18 (45%)	0.64
Myocardial calfication on CT		7 (20.6%)	5 (12.5%)	0.37
Diffuse scar^b^		25 (49.0%)	39 (57.3%)	0.46
Intramural scar		4 (7.7%)	9 (13.2%)	0.39

*Note:* Patients' demographic characteristics were compared at baseline between the elective and urgent groups. Continuous data was represented by the mean and standard deviation for normal distributions, and with the median and interquartile range (IQR) for non‐normal distributions. Categorical variables were reported as percentages.

Abbreviations: CT, computer tomography; MRI, magnetic resonance imaging.

^a^ Statistically significant.

^b^ Diffuse scar is defined as scar larger than territory within one coronary distribution.

Procedural characteristics between our two groups were compared (Table 2). The urgent group more frequently were under general anesthesia (38.2% vs. 21.2%, *p* = 0.047) at the time of ablation. Three patients were intubated for their ES before ablation in the urgent group compared to none in the elective group (*p* = 0.26). There were four patients who required extracorporeal membrane oxygenation (ECMO) support in the urgent compared to none in elective group. There were no statistically significant differences in any of the other characteristics including the number of inducible VTs, VT inducibility rates, procedure time and radiofrequency ablation time.

**Table 2:** Procedural characteristics.

Characteristic	Subcategory	Elective	Urgent	*p* value
Number of inducible VTs		3.5 (IQR 2–7)	4 (IQR 2–8)	0.17
Preoperatively intubated		0 (0%)	3 (4.4%)	0.26
Preoperative pressor use		0 (0%)	2 (2.9%)	0.5
Intraoperative pressor use		12 (23.1%)	18 (26.4%)	0.83
ECMO intraoperative use		0 (0%)	4 (5.9%)	0.13
tMCS intraoperative use		1 (1.92%)	1 (1.47%)	0.99
General anesthesia		11 (21.2%)	26 (38.2%)	0.047^a^
Total intra‐Op fluids (mL)		2700 (IQR 2033–3400)	2800 (IQR 2125–3748)	0.51
Radiofrequency ablation time (min)		93 (IQR 59–128)	99 (IQR 60–157)	0.39
Procedure time (min)		350 (IQR 298–432)	348 (IQR 264–502)	0.89
Fluoroscopy time (min)		24 (IQR– 1442)	21 (IQR 14‐41)	0.63
Epicardial ablation performed		5 (9.6%)	11 (16.2%)	0.42
Clinical VT inducibility		*n* = *44*	*n* = *54*	0.61
	Yes	37	43	
	No	7	11	
Difficulty mapping^b^		34 (65.4%)	41 (60.3%)	0.7

*Note:* Intraprocedural characteristics in the elective and urgent groups are presented below. Mapping difficulty was subjectively assessed by the operator and was typically due to VT non inducibility, hemodynamic instability, or intramural VT origin. Continuous data was represented by the mean and standard deviation for normal distributions, and with the median and interquartile range (IQR) for non‐normal distributions. Categorical variables were reported as percentages

Abbreviations: ECMO, extracorporeal membrane oxygenationt; MCS, temporary mechanical circulatory support (intra‐aortic balloon pump or Impella); VT, ventricular tachycardia.

^a^ Statistically significant.

^b^ Difficulty mapping includes the following factors: Hemodynamically unstable patients, difficulty inducing the ventricular arrhythmia, intramural origin site, papillary muscle origin site, need for epicardial access, difficulty establishing catheter contact.

**Figure 1:**
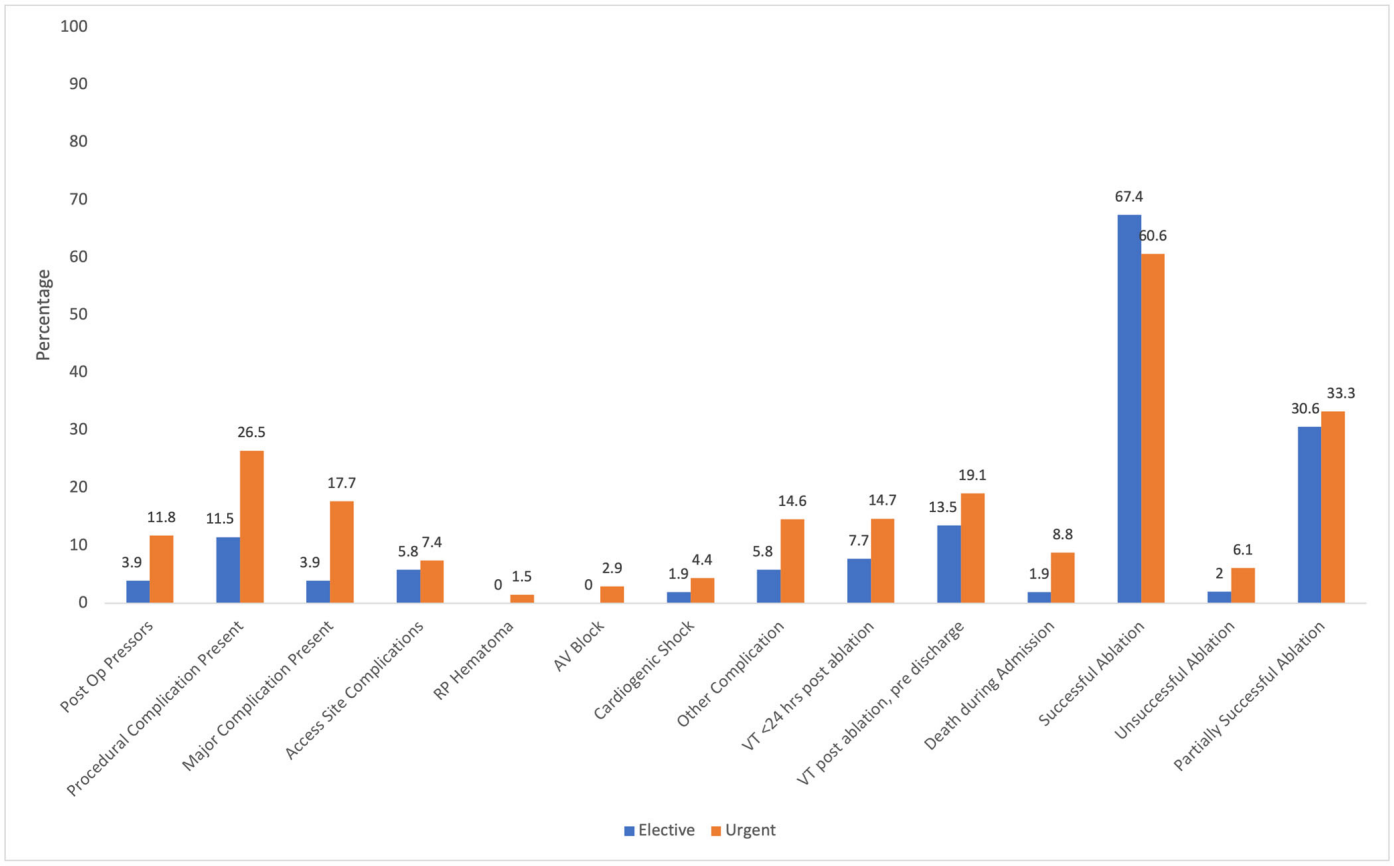
Immediate postoperative outcomes. Complication rates post ablation and before discharge were compared between the elective and urgent groups. Categorical variables were reported as percentages. Major complications included cardiac arrest, death, cardiogenic shock, post procedural septic shock, post procedural hypoxic respiratory failure, postprocedural hemorrhagic shock, cardiac perforation, and complete AV block. Ablation was deemed successful if all inducible VT were ablated. Partial success was defined as ablation of clinical VT but with lingering nonclinical VT at the end of the case. Unsuccessful ablation was defined as failure to ablate clinical VT. Urgent group had statistically higher rate of major complications; urgent group trended toward more overall complications and less procedural success but the differences were not statistically significant. *Statistically significant.

The immediate postprocedural outcomes were compared (Figure 1). Major postoperative complications during index hospitalization were significantly more common in the urgent group (17.7% vs. 3.9%, *p* = 0.023). Other postoperative complications, including overall complication rate, VT recurrence in 24 h, and death during index admission, were higher in the urgent group but the differences were not statistically significant. Acute procedural success was achieved in 67.4% of patients in elective group, compared with 60.6% in the urgent group (*p* = 0.55).

Long term survival (Central Illustration 1) was significantly longer in the elective ablation group at time of last follow up compared to the urgent group (*χ* ^2^ = 7.4, *p* = 0.0065). There was no significant difference in VT‐free survival (*χ* ^2^ = 1.3, *p* = 0.26). Similarly, ES‐free recurrence and ablation free survival were similar across both groups, (*χ* ^2^ = 0.99, *p* = 0.32) and (*χ* ^2^ = 1.1, *p* = 0.29), respectively.

**Central Illustration 1:**
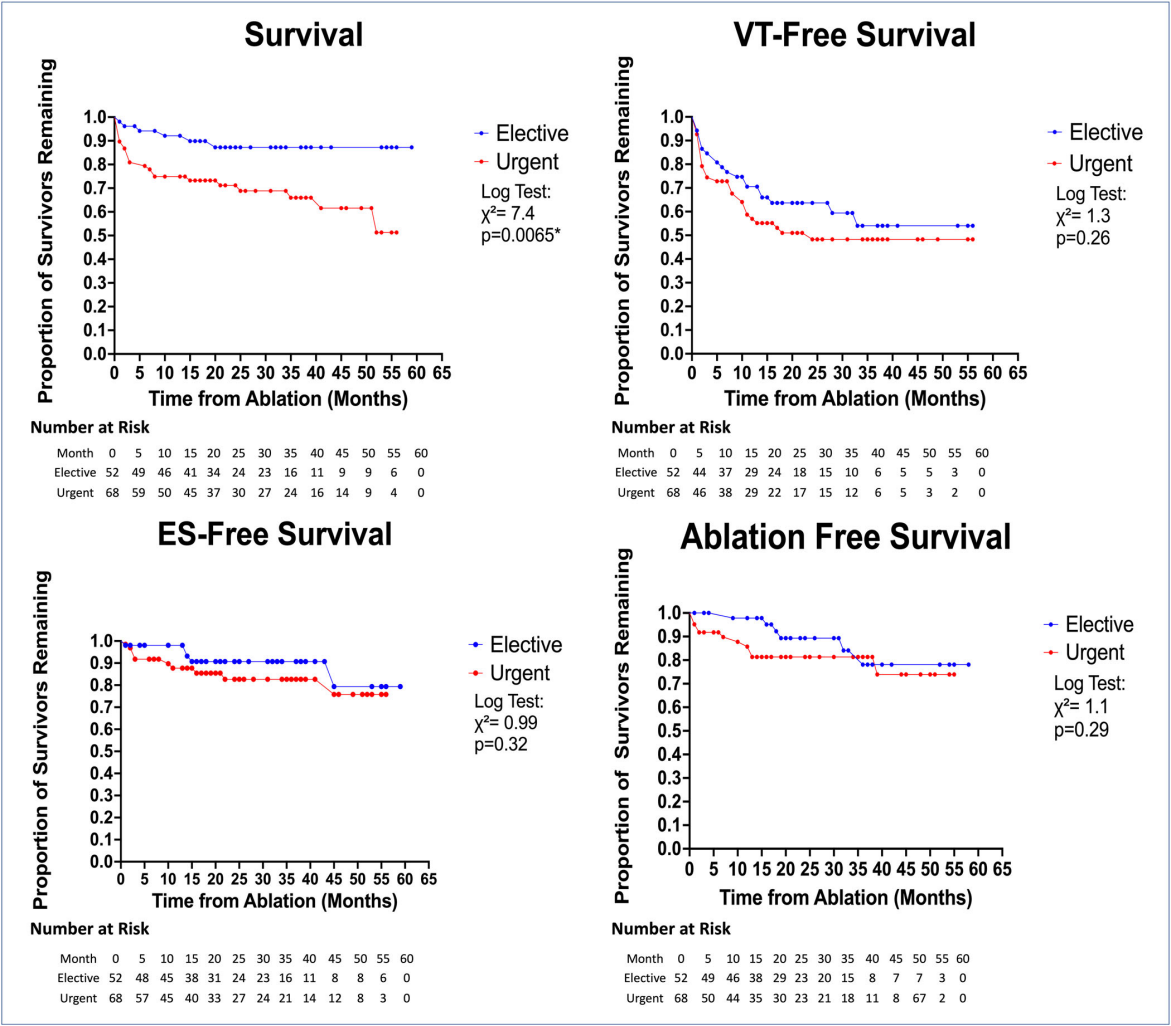
Kaplan–Meir survival curves comparing the elective and urgent groups from time of ablation. Top Left: Long‐term survival. Top Right: VT‐free survival. Bottom Left: ES‐free survival. Bottom Right: Repeat ablation‐free survival. Long term survival was significantly improved in the elective group. No significant difference was identified in the remaining outcomes.

Cox proportional hazards model identified numerous independent predictors of survival (Table 3) and VT‐free survival (Table 4). In terms of survival, the need for urgent ablation was predictive of increased mortality in both the univariate and multivariate analyses (*p* = 0.016 and *p* = 0.039, respectively). The univariate analysis suggested that age, high PAINESD score, presence of ≥ stage 3 chronic kidney disease, reduced left ventricular ejection fraction, need for intraoperative pressors, history of prior arrest and number of inducible VTs were all predictive of increased mortality. However, after accounting for confounders through the multivariate model, only the need for urgent ablation and the number of inducible VTs intraoperatively (*p* = 0.0035) predicted higher mortality. In terms of VT‐free survival, urgent ablation was not independently associated with increased VT recurrence (*p* = 0.88). However, the use of beta blockers was significantly associated with reduced VT recurrence (*p* = 0.0003) in the multivariate analysis.

**Table 3:** Cox proportional hazards model for mortality.

Variable	Univariate hazard ratio	Univariate *p* value	Multivariate hazard ratio	Multivariate *p* value
Urgent ablation	2.66	0.016^a^	3.66	0.039^a^
Age	1.04	0.028^a^	1.02	0.58
Female	1.93	0.15	2.4	0.21
PAINESD	1.08	0.0033^a^	1.23	0.022
Body Mass Index	0.97	0.88	0.97	0.46
Hypertension	2.05	0.18	2.9	0.16
Diabetes mellitus	1.45	0.3	2.41	0.18
Chronic kidney disease, ≥ stage 3	3.18	0.009^a^	0.93	0.14
Chronic obstructive pulmonary disease	1.1	0.85	0.31	0.22
Ischemic cardiomyopathy	1.12	0.75	0.25	0.27
Nonischemic cardiomyopathy	0.86	0.68	1.39	0.79
Left ventricular ejection fraction	0.93	< 0.0001	0.96	0.1
Left ventricular assist device	1.7	0.28	1.44	0.59
Atrial fibrillation	1.13	0.74	0.64	0.37
Prior arrest	2.16	0.038^a^	1.27	0.22
Prior VT ablation	1.55	0.21	1.22	0.7
Cerebrovascular accident	1.2	0.7	0.45	0.44
On ≥ 2 antiarrhythmic drugs	1.81	0.16	0.93	0.37
Beta blocker	1.18	0.76	0.47	0.3
Preoperative pressors	7.91	0.18	0.96	0.98
Intraoperative pressors	2.49	0.014^a^	2.8	0.056
General anesthesia	1.38	0.38	0.43	0.22
Epicardial ablation	1.02	0.96	0.45	0.34
Number of inducible VT	1.16	< 0.0001^a^	1.15	0.0035^a^
Intramural scar	1.24	0.66	1.43	0.6576
Diffuse scar^b^	1.07	0.84	1.87	0.2162
Difficulty mapping^c^	1.08	0.83	1.73	0.3325

*Note:* Cox proportional hazards model was employed to identify univariate hazard ratios and *p* values for each independent variable compared to mortality, as well as a multivariate hazard ratio that accounts for all the other variables in this table. The need for urgent ablation as well as a higher number of inducible VTs intraoperatively were associated with a statistically significant rise in mortality in the multivariable model.

Abbreviation: VT, ventricular tachycardia.

^a^ Statistically significant.

^b^ Diffuse scar is defined as scar larger than territory within one coronary distribution.

^c^ Difficulty mapping includes the following factors: Hemodynamically unstable patients, difficulty inducing the ventricular arrhythmia, intramural origin site, papillary muscle origin site, need for epicardial access, difficulty establishing catheter contact.

**Table 4:** Cox proportional hazards model for ventricular tachycardia recurrence.

Variable	Univariate hazard ratio	Univariate *p* value	Multivariate hazard ratio	Multivariate *p* value
Urgent ablation	1.35	0.3	1.06	0.88
Age	1.01	0.3	0.99	0.75
Female	1.2	0.66	0.88	0.83
PAINESD	1.03	0.093	1.05	0.41
Body Mass Index	0.98	0.43	0.96	0.15
Hypertension	1.02	0.96	1.18	0.75
Diabetes mellitus	1.18	0.52	1.62	0.27
Chronic kidney disease, ≥ stage 3	1.96	0.02	1.41	0.41
Chronic obstructive pulmonary disease	1.84	0.084	1.85	0.3
Ischemic cardiomyopathy	0.7	0.21	0.11	0.065
Nonischemic cardiomyopathy	1.25	0.42	0.33	0.35
Left ventricular ejection fraction	0.98	0.036^a^	0.99	0.32
Left ventricular assist device	1.02	0.97	0.68	0.5
Atrial fibrillation	1.27	0.41	1.18	0.66
Prior arrest	1.97	0.032	1.38	0.43
Prior VT ablation	1.39	0.24	1.78	0.12
Cerebrovascular accident	0.99	0.99	0.72	0.55
On ≥ 2 antiarrhythmic drugs	1.22	0.52	1.07	0.86
Beta blocker	0.51	0.055	0.19	0.0003^a^
Preoperative pressors	1.27	0.82	1.75	0.65
Intraoperative pressors	1.4	0.29	2.3	0.055
General anesthesia	1.22	0.52	0.7	0.42
Epicardial ablation	1.41	0.35	1.19	0.77
Number of inducible VT	1.08	0.0054^a^	1.04	0.29
Intramural scar	1	0.99	1.01	0.99
Diffuse scar^b^	0.99	0.97	0.6	0.13
Difficulty mapping^c^	0.98	0.93	1.12	0.77

*Note:* Cox proportional hazards model was employed to identify univariate hazard ratios and *p* values for each independent variable compared to ventricular tachycardia (VT) recurrence, as well as a multivariate hazard ratio that accounts for all the other variables in this table. The use of beta‐blockers was the only significant variable predictive of reduced VT recurrence in the multivariable model. The need for urgent ablation did not predict increased recurrence.

^a^ Statistically significant.

^b^ Diffuse scar is defined as scar larger than territory within one coronary distribution.

^c^ Difficulty mapping includes the following factors: Hemodynamically unstable patients, difficulty inducing the ventricular arrhythmia, intramural origin site, papillary muscle origin site, need for epicardial access, difficulty establishing catheter contact.

We then compared causes of mortality between our two groups (Supporting Information S3: Table 3). Significantly more patients died of a ventricular arrhythmia in the urgent group compared to the elective group (17.6% vs. 3.9%, *p* = 0.023).

## 4 Discussion

In this study, patients with ES who were able to be stabilized on AAD and subsequently underwent VT ablation electively had improved long term survival compared to patients who were not able to be stabilized on AAD and required urgent ablation. This relationship persisted after multivariate regression. Furthermore, there appeared to be a significant increase in major post‐procedural complications in the urgent group. There were however no significant differences in VT‐free, ES‐free, or repeat VT‐ablation free survival.

Several randomized controlled trials have brought forth the superior efficacy and safety of catheter ablation in management of ventricular arrhythmias. In the VANISH trial, catheter ablation was found to have improved VA burden as compared to escalation of AAD [23]. The SURVIVE‐VT and VANISH 2 trials later demonstrated that catheter ablation as an initial management strategy had improved primary composite cardiovascular outcomes when compared to initial medical therapy, although there was no significant effect on mortality [24, 25]. The evidence of VT ablation is further supported in a meta‐analysis of randomized controlled trials of ablative vs nonablative strategies for VA [26].

Within the context of ES, the evidence is also supportive of catheter ablation. A multicenter retrospective analysis focused on ES revealed reduced VT recurrence and improved survival in patients who underwent catheter ablation compared to medical therapy [27]. The timing of intervention is less defined, however. A retrospective analysis by Huang et al compared early ablation during the initial hospitalization with ES to initial medical therapy with delayed ablation [34]. The study suggested that early ablation was associated with improved VT‐free survival and reduced hospitalizations but found no significant differences in mortality. This result was surprising as it was previously expected that VT ablation may be less successful during the inflammatory state surrounding ES based on prior studies that highlighted how patients with ES had higher in hospital mortality following VT ablation compared to patients who had VT but not ES [35]. More recently, a study compared outcomes of patients who underwent immediate ablation following ED admission with VT to patients who underwent elective ablation. Although not restricted to ES as with our study, the authors showed that VT ablation following ED admission was associated with increased in‐hospital mortality, and increased periprocedural complications (largely thromboembolic and infectious) [36]. Patients admitted from the ED were unsurprisingly sicker at baseline, but impact of immediate ablation at time of ED admission was independently associated with these outcomes. These findings are in congruence with our results and suggest that the need for an urgent ablation is predictive of a worse prognosis, not only in ES but for VA in general.

There are two categories of ES: those that can be stabilized on AAD and those that are intractable and refractory to AAD. This current study showed that the intractable ES had worse long‐term survival despite having a shorter time between diagnosis and ablation—the urgent group had their ablation 7 days postepisode of ES, compared to 86 days in the elective group. While previous studies showed improved survival among those with ES that underwent early ablation, this study suggests that the intractability of the ES is an important prognostic factor that should be considered during triage.

The survival difference appears to be partially driven by an increased rate of deaths during the index hospitalizations, possibly from immediate post‐procedural complications (Figure 1). An inspection of the survival curve (Central Illustration 1) highlights an immediate survival gap. However, the persistence of the survival benefit years after the ablation suggests that there continues to be a long‐term benefit. Although the VT‐free survival was similar between the two groups, it does appear that the survival benefit is partially explained by increased mortality from recurrent VTs (Supporting Information S3: Table 3). This could suggest that although VT recurrence rates were similar, the patients in the urgent group who did have recurrent or persistent VT had the arrhythmia at a higher burden that may be contributory to increased mortality. Many patients in both groups died of non‐VT associated factors, including heart failure, infection, malignancy, and so forth. It should be mentioned that the patients in the elective group were more likely to have preprocedural Cardiac MRI and CTA although the difference was not statistically significant. It is well established that the utility of image integration is associated with improved ablation efficacy [37, 38]. In our study, the elective group did have a higher rate of successful ablation, but the difference was not significant. It warrants mention that the imaging findings did not typically influence operators' decision on urgency of ablation, but rather may have increased likelihood for pursuing ablation in the first place especially if the scar appeared localized or easily accessible. In some cases, if the imaging suggested epicardial access was needed, timing of ablation may have been adjusted to accommodate an operator experienced in that form of access. Lastly, if an intracardiac thrombus was identified, then ablation would be delayed.

Our study had several important limitations. First, the single center nature of this study limits its external validity, as institutional practices of our hospital may not entirely represent what is practiced elsewhere. Second, as a retrospective study, we are unable to establish causality despite controlling for many variables with multi‐variate regression and it is possible some confounding may persist. Given the inherent stability of the elective group, it is possible that the observed survival benefit reflects stability rather than AAD responsiveness. Third, we did have several patients who were lost to follow‐up after several months; these patients were censored during the survival analysis, but it is possible some of the conclusions may be different had they continued to follow up. Fourth, although a large sample size for patients with ES in a single center, our study may have been underpowered to detect certain differences. Lastly, it is important to not interpret the findings of this study within the context of early versus delayed ablation strategy, as our groups were divided based on need for urgent ablation for clinical reasons vs those who could be stabilized.

## 5 Conclusion

Patients with ES who were successfully stabilized on oral AAD before undergoing a VT ablation electively had improved postablation survival compared to patients who were not stabilized on AAD and required urgent VT ablation. This suggests the intractability of the ES is an important prognostic factor that should be considered during triage. Future prospective studies (ideally randomized controlled trials) will be helpful to determine the role of early versus delayed VT ablation for ES.

## Conflicts of Interest

The authors declare no conflicts of interest.

## Supporting information


**Table Supplemental 1:** Antiarrhythmic Drug (AAD) Utilization Rates in Elective vs Urgent groups. Overall, no significant differences in the utilization of any AAD between either group.

**Table Supplemental 2:** Pre‐procedural AAD holding rates between Elective vs Urgent groups. Overall, no significant differences in the rates of holding any AAD between either group.

**Table Supplemental 3:** Cause of death comparison between Elective vs Urgent groups. Ventricular arrhythmia associated deaths were significantly higher in the urgent group.

supmat.

## Data Availability

The data that support the findings of this study are available from the corresponding author upon reasonable request.

## References

[1] I. Elsokkari and J. L. Sapp , “Electrical Storm: Prognosis and Management,” Progress in Cardiovascular Diseases 66 (May/June 2021): 70–79, 10.1016/j.pcad.2021.06.007. 34332662

[2] A. Verma , F. Kilicaslan , N. F. Marrouche , et al., “Prevalence, Predictors, and Mortality Significance of the Causative Arrhythmia in Patients With Electrical Storm,” Journal of Cardiovascular Electrophysiology 15, no. 11 (2004): 1265–1270, 10.1046/j.1540-8167.2004.04352.x. 15574176

[3] F. Guerra , M. Shkoza , L. Scappini , M. Flori , and A. Capucci , “Role of Electrical Storm as a Mortality and Morbidity Risk Factor and Its Clinical Predictors: A Meta‐Analysis,” Europace : European Pacing, Arrhythmias, and Cardiac Electrophysiology : Journal of the Working Groups on Cardiac Pacing, Arrhythmias, and Cardiac Cellular Electrophysiology of the European Society of Cardiology 16, no. 3 (2014): 347–353, 10.1093/europace/eut304. 24096960

[4] I. Elsokkari , R. Parkash , A. Tang , et al., “Mortality Risk Increases With Clustered Ventricular Arrhythmias in Patients With Implantable Cardioverter‐Defibrillators,” JACC: Clinical Electrophysiology 6, no. 3 (March 2020): 327–337, 10.1016/j.jacep.2019.11.012. 32192684

[5] D. Bänsch , D. Böcker , J. Brunn , M. Weber , G. Breithardt , and M. Block , “Clusters of Ventricular Tachycardias Signify Impaired Survival In Patients With Idiopathic Dilated Cardiomyopathy and Implantable Cardioverter Defibrillators,” Journal of the American College of Cardiology 36, no. 2 (August 2000): 566–573, 10.1016/s0735-1097(00)00726-9. 10933373

[6] D. V. Exner , S. L. Pinski , D. G. Wyse , et al., “Electrical Storm Presages Nonsudden Death: The Antiarrhythmics Versus Implantable Defibrillators (AVID) Trial,” Circulation 103, no. 16 (April 2001): 2066–2071, 10.1161/01.cir.103.16.2066. 11319196

[7] R. G. Trohman , “Etiologies, Mechanisms, Management, and Outcomes of Electrical Storm,” Journal of Intensive Care Medicine 39, no. 2 (2024): 99–117, 10.1177/08850666231192050. 37731333

[8] B. Dinov , A. Darma , S. Nedios , and G. Hindricks , “Management of Patients With Electrical Storm: An Educational Review,” European Heart Journal. Acute Cardiovascular Care 12, no. 1 (2023): 69–73, 10.1093/ehjacc/zuac160. 36574428

[9] S. J. Connolly , “Comparison of Beta‐Blockers, Amiodarone Plus Beta‐Blockers, or Sotalol for Prevention of Shocks From Implantable Cardioverter Defibrillators: The Optic Study: A Randomized Trial,” Journal of the American Medical Association 295, no. 2 (2006): 165–171, 10.1001/jama.295.2.165. 16403928

[10] P. Dorian , D. Cass , B. Schwartz , R. Cooper , R. Gelaznikas , and A. Barr , “Amiodarone as Compared With Lidocaine for Shock‐Resistant Ventricular Fibrillation,” New England Journal of Medicine 346, no. 12 (2002): 884–890, 10.1056/NEJMoa013029. 11907287

[11] S. Güler , H. Könemann , J. Wolfes , et al., “Lidocaine as an Anti‐Arrhythmic Drug: Are There Any Indications Left?,” Clinical and Translational Science 16, no. 12 (2023): 2429–2437, 10.1111/cts.13650. 37781966 PMC10719458

[12] A. Pacifico , S. H. Hohnloser , J. H. Williams , et al., “Prevention of Implantable‐Defibrillator Shocks by Treatment With Sotalol,” New England Journal of Medicine 340, no. 24 (1999): 1855–1862, 10.1056/NEJM199906173402402. 10369848

[13] M. Ortiz , A. Martín , F. Arribas , et al., “Randomized Comparison of Intravenous Procainamide vs. Intravenous Amiodarone for the Acute Treatment of Tolerated Wide QRS Tachycardia: The PROCAMIO Study,” European Heart Journal 38, no. 17 (2017): 1329–1335, 10.1093/eurheartj/ehw230. 27354046 PMC5410924

[14] M. Toniolo , D. Muser , G. Mugnai , et al., “Comparison of Oral Procainamide and Mexiletine Treatment of Recurrent and Refractory Ventricular Tachyarrhythmias,” Journal of Clinical Medicine 13, no. 20 (2024): 6099, 10.3390/jcm13206099. 39458049 PMC11508758

[15] J. Larson , L. Rich , A. Deshmukh , E. C. Judge , and J. J. Liang , “Pharmacologic Management for Ventricular Arrhythmias: Overview of Anti‐Arrhythmic Drugs,” Journal of Clinical Medicine 11, no. 11 (2022): 3233, 10.3390/jcm11113233. 35683620 PMC9181251

[16] S. Chatzidou , C. Kontogiannis , D. I. Tsilimigras , et al., “Propranolol Versus Metoprolol for Treatment of Electrical Storm in Patients With Implantable Cardioverter‐Defibrillator,” Journal of the American College of Cardiology 71, no. 17 (2018): 1897–1906, 10.1016/j.jacc.2018.02.056. 29699616

[17] J. S. Bundgaard , P. K. Jacobsen , J. Grand , et al., “Deep Sedation as Temporary Bridge to Definitive Treatment of Ventricular Arrhythmia Storm,” European Heart Journal. Acute Cardiovascular Care 9, no. 6 (2020): 657–664, 10.1177/2048872620903453. 32193944

[18] M. Fudim , Y. J. Qadri , N. H. Waldron , et al., “Stellate Ganglion Blockade for the Treatment of Refractory Ventricular Arrhythmias,” JACC: Clinical Electrophysiology 6, no. 5 (2020): 562–571, 10.1016/j.jacep.2019.12.017. 32439042

[19] M. Fudim , R. Boortz‐Marx , A. Ganesh , et al., “Stellate Ganglion Blockade for the Treatment of Refractory Ventricular Arrhythmias: A Systematic Review and Meta‐Analysis,” Journal of Cardiovascular Electrophysiology 28, no. 12 (2017): 1460–1467, 10.1111/jce.13324. 28833780

[20] D. H. Do , J. Bradfield , O. A. Ajijola , et al., “Thoracic Epidural Anesthesia Can Be Effective for the Short‐Term Management of Ventricular Tachycardia Storm,” Journal of the American Heart Association 6, no. 11 (2017): e007080, 10.1161/JAHA.117.007080. 29079570 PMC5721785

[21] M. Vaseghi , J. Gima , C. Kanaan , et al., “Cardiac Sympathetic Denervation in Patients With Refractory Ventricular Arrhythmias or Electrical Storm: Intermediate and Long‐Term Follow‐Up,” Heart Rhythm : The Official Journal of the Heart Rhythm Society 11, no. 3 (2014): 360–366, 10.1016/j.hrthm.2013.11.028. PMC4253031 24291775

[22] B. Curtis , G. VanAken , M. Al‐Sadawi , et al., “Safety and Outcomes of Surgical Cardiac Sympathetic Denervation When Used as Salvage Therapy Among High‐Risk Patients With Refractory Ventricular Arrhythmias,” Journal of Interventional Cardiac Electrophysiology 68, no. 2 (2025): 411–413, 10.1007/s10840-024-01874-z. 39007967

[23] J. L. Sapp , G. A. Wells , R. Parkash , et al., “Ventricular Tachycardia Ablation versus Escalation of Antiarrhythmic Drugs,” New England Journal of Medicine 375, no. 2 (2016): 111–121, 10.1056/NEJMoa1513614. 27149033

[24] Á. Arenal , P. Ávila , J. Jiménez‐Candil , et al., “Substrate Ablation vs Antiarrhythmic Drug Therapy for Symptomatic Ventricular Tachycardia,” Journal of the American College of Cardiology 79, no. 15 (2022): 1441–1453, 10.1016/j.jacc.2022.01.050. 35422240

[25] J. L. Sapp , A. S. L. Tang , R. Parkash , et al., “Catheter Ablation or Antiarrhythmic Drugs for Ventricular Tachycardia,” New England Journal of Medicine 392, no. 8 (2025): 737–747, 10.1056/NEJMoa2409501. 39555820

[26] D. Patel , V. Hasselblad , K. P. Jackson , S. D. Pokorney , J. P. Daubert , and S. M. Al‐Khatib , “Catheter Ablation for Ventricular Tachycardia (VT) in Patients With Ischemic Heart Disease: A Systematic Review and a Meta‐Analysis of Randomized Controlled Trials,” Journal of Interventional Cardiac Electrophysiology 45, no. 2 (2016): 111–117, 10.1007/s10840-015-0083-4. 26695501

[27] K. Benali , S. Ninni , C. Guenancia , et al., “Impact of Catheter Ablation of Electrical Storm on Survival: A Propensity Score‐Matched Analysis,” JACC Clin Electrophysiol (July 2024), 10.1016/j.jacep.2024.05.032. 39093275

[28] M. Vaseghi , T. Y. Hu , R. Tung , et al., “Outcomes of Catheter Ablation of Ventricular Tachycardia Based on Etiology in Nonischemic Heart Disease,” JACC: Clinical Electrophysiology 4, no. 9 (2018): 1141–1150, 10.1016/j.jacep.2018.05.007. 30236386 PMC6242273

[29] D. Muser , J. J. Liang , R. K. Pathak , et al., “Long‐Term Outcomes of Catheter Ablation of Electrical Storm in Nonischemic Dilated Cardiomyopathy Compared With Ischemic Cardiomyopathy,” JACC: Clinical Electrophysiology 3, no. 7 (2017): 767–778, 10.1016/j.jacep.2017.01.020. 29759543

[30] B. Dinov , L. Fiedler , R. Schönbauer , et al., “Outcomes In Catheter Ablation of Ventricular Tachycardia in Dilated Nonischemic Cardiomyopathy Compared With Ischemic Cardiomyopathy: Results From the Prospective Heart Centre of Leipzig VT (HELP‐VT) Study,” Circulation 129, no. 7 (2014): 728–736, 10.1161/CIRCULATIONAHA.113.003063. 24211823

[31] B. Dinov , A. Arya , L. Bertagnolli , et al., “Early Referral for Ablation of Scar‐Related Ventricular Tachycardia Is Associated With Improved Acute and Long‐Term Outcomes: Results From the Heart Center of Leipzig Ventricular Tachycardia Registry,” Circulation. Arrhythmia and Electrophysiology 7, no. 6 (2014): 1144–1151, 10.1161/CIRCEP.114.001953. 25262159

[32] D. S. Frankel , S. E. Mountantonakis , M. R. Robinson , E. S. Zado , D. J. Callans , and F. E. Marchlinski , “Ventricular Tachycardia Ablation Remains Treatment of Last Resort in Structural Heart Disease: Argument for Earlier Intervention,” Journal of Cardiovascular Electrophysiology 22, no. 10 (2011): 1123–1128, 10.1111/j.1540-8167.2011.02081.x. 21539642

[33] J. Romero , L. Di Biase , J. C. Diaz , et al., “Early Versus Late Referral for Catheter Ablation of Ventricular Tachycardia in Patients With Structural Heart Disease,” JACC: Clinical Electrophysiology 4, no. 3 (2018): 374–382, 10.1016/j.jacep.2017.12.008. 30089564

[34] K. Huang , R. G. Bennett , T. Campbell , et al., “Early Catheter Ablation Versus Initial Medical Therapy for Ventricular Tachycardia Storm,” Circulation: Arrhythmia and Electrophysiology 15, no. 12 (2022): e011129, 10.1161/CIRCEP.122.011129. 36399370

[35] P. Vergara , R. Tung , M. Vaseghi , et al., “Successful Ventricular Tachycardia Ablation in Patients With Electrical Storm Reduces Recurrences and Improves Survival,” Heart Rhythm: Official Journal of the Heart Rhythm Society 15, no. 1 (2018): 48–55, 10.1016/j.hrthm.2017.08.022. 28843418

[36] J. Dickow , N. Gessler , O. Anwar , et al., “Safety of Immediate Catheter Ablation of Ventricular Arrhythmias in Patients Admitted via the Emergency Department,” Journal of Interventional Cardiac Electrophysiology 68 (February 2025): 1257–1266, 10.1007/s10840-025-02020-z. 40019686

[37] K. C. Siontis , H. M. Kim , G. Sharaf Dabbagh , et al., “Association of Preprocedural Cardiac Magnetic Resonance Imaging With Outcomes of Ventricular Tachycardia Ablation in Patients With Idiopathic Dilated Cardiomyopathy,” Heart Rhythm: Official Journal of the Heart Rhythm Society 14, no. 10 (2017): 1487–1493, 10.1016/j.hrthm.2017.06.003. 28603002

[38] D. Soto ‐Iglesias , D. Penela , B. J áuregui , et al., “Cardiac Magnetic Resonance‐Guided Ventricular Tachycardia Substrate Ablation,” JACC. Clinical Electrophysiology 6, no. 4 (2020): 436–447, 10.1016/j.jacep.2019.11.004. 32327078

