# Supplementary material for: Prognostic Value of Antiarrhythmic Drug Suppression of Electrical Storm Prior to Ventricular Tachycardia Ablation
Source: J Cardiovasc Electrophysiol. 2025 Oct 15;36(12):3310–20. doi: 10.1111/jce.70133 (PMC12697235; doi:10.1111/jce.70133)
Supplement: Supplementary file 2 — Table Supplemental 2: Pre‐procedural AAD holding rates between Elective vs Urgent groups. Overall, no significant differences in the rates of holding any AAD between either group. [file JCE-36-3310-s003.docx]

**Table Supplemental 2:**

Pre-procedural AAD holding rates between Elective vs Urgent groups. Overall, no significant differences in the rates of holding any AAD between either group.

| **Variable** | **Elective** | **Urgent** | **P-Value** |
| --- | --- | --- | --- |
| Amiodarone held pre-op | 84.40% | 76.20% | 0.72 |
| Mexiletine held pre-op | 95% | 97.80% | >0.99 |
| Sotalol held pre-op | 90% | 88.90% | >0.99 |
