# Supplementary material for: Prognostic Value of Antiarrhythmic Drug Suppression of Electrical Storm Prior to Ventricular Tachycardia Ablation
Source: J Cardiovasc Electrophysiol. 2025 Oct 15;36(12):3310–20. doi: 10.1111/jce.70133 (PMC12697235; doi:10.1111/jce.70133)
Supplement: Supplementary file 3 — Table Supplemental 3: Cause of death comparison between Elective vs Urgent groups. Ventricular arrhythmia associated deaths were significantly higher in the urgent group. [file JCE-36-3310-s001.docx]

**Table Supplemental 3:**

Cause of death comparison between Elective vs Urgent groups. Ventricular arrhythmia associated deaths were significantly higher in the urgent group.

| **Cause of Death** | **Elective** | **Urgent** | **P-Value** |
| --- | --- | --- | --- |
| Ventricular Arrythmia | 3.9% (2) | 17.6% (12) | 0.023 |
| Other | 3.9% (2) | 8.8% (6) | 0.46 |
| Unknown | 3.9% (2) | 7.3% (5) | 0.7 |
