# Supplementary material for: Prognostic Value of Antiarrhythmic Drug Suppression of Electrical Storm Prior to Ventricular Tachycardia Ablation
Source: J Cardiovasc Electrophysiol. 2025 Oct 15;36(12):3310–20. doi: 10.1111/jce.70133 (PMC12697235; doi:10.1111/jce.70133)
Supplement: Supplementary file 4 — supmat. [file JCE-36-3310-s004.docx]

**Table Supplemental 1:**

Antiarrhythmic Drug (AAD) Utilization Rates in Elective vs Urgent groups. Overall, no significant differences in the utilization of any AAD between either group.

Dosing:

- Amiodarone was started as an IV in the acute setting and loaded until steady state with 10g has been achieved, with oral maintenance doses ranging from 200 to 400 mg a day.
- Lidocaine was typically started acutely at 1-1.5 mg/kg, with maintenance infusion dosing being adjusted based on lidocaine levels.
- Mexiletine was used in outpatient setting at 150-200 mg three times a day.
- Procainamide was usually loaded with 20 mg/min for 1000 mg bolus before transition to 1-4 mg/min maintenance, and further adjusted by procainamide level.
- Quinidine was typically dosed at 200-300 mg three times a day, but in some patients as high as 400 mg every 6 hours.
- Sotalol dosing was maximized as tolerated by Qtc during the loading phase, as high as 160 mg twice daily.

**Table Supplemental 2:**

Pre-procedural AADs holding rates between Elective vs Urgent groups. Overall, no significant differences in the rates of holding any AADs between either group.

**Table Supplemental 3:**

Cause of death comparison between Elective vs Urgent groups. Ventricular arrhythmia associated deaths were significantly higher in the urgent group.
